# Dietary habits in three Central and Eastern European countries: the HAPIEE study

**DOI:** 10.1186/1471-2458-9-439

**Published:** 2009-12-01

**Authors:** Sinéad Boylan, Ailsa Welch, Hynek Pikhart, Sofia Malyutina, Andrzej Pajak, Ruzena Kubinova, Oksana Bragina, Galina Simonova, Urszula Stepaniak, Aleksandra Gilis-Januszewska, Lubomíra Milla, Anne Peasey, Michael Marmot, Martin Bobak

**Affiliations:** 1 Department of Epidemiology and Public Health, University College London, London, UK; 2 School of Medicine, Health Policy and Practice, University of East Anglia, Norwich, UK; 3 Institute of Internal Medicine, Russian Academy of Medical Sciences, Novosibirsk, Russia; 4 Department of Epidemiology and Population Studies, Jagiellonian University Medical College, Krakow, Poland; 5 National Institute of Public Health, Prague, Czech Republic

## Abstract

**Background:**

The high cardiovascular mortality in Eastern Europe has often been attributed to poor diet, but individual-level data on nutrition in the region are generally not available. This paper describes the methods of dietary assessment and presents preliminary findings on food and nutrient intakes in large general population samples in Russia, Poland and the Czech Republic.

**Methods:**

The HAPIEE (Health, Alcohol and Psychosocial factors In Eastern Europe) study examined random samples of men and women aged 45-69 years at baseline in Novosibirsk (Russia), Krakow (Poland) and six Czech urban centres in 2002-2005. Diet was assessed using a food frequency questionnaire (at least 136 items); complete dietary information was available for 26,870 persons.

**Results:**

Total energy intakes among men ranged between 8.7 MJ in the Czech sample and 11.7 MJ in the Russian sample, while among women, energy intakes ranged between 8.2 MJ in the Czech sample and 9.8 MJ in the Russian sample. A Healthy Diet Indicator (HDI), ranging from a score of 0 (lowest) to 7 (highest), was developed using the World Health Organisation's (WHO) guidelines for the prevention of chronic diseases. The mean HDI scores were low, ranging from 1.0 (SD = 0.7) among the Polish subjects to 1.7 (SD = 0.8) among the Czech females. Very few subjects met the WHO recommended intakes for complex carbohydrates, pulses or nuts; intakes of saturated fatty acids, sugar and protein were too high. Only 16% of Polish subjects met the WHO recommendation for polyunsaturated fat intake. Consumption of fruits and vegetables was lower than recommended, especially among those Russian subjects who were assessed during the low intake season. Fewer than 65% of subjects consumed adequate amounts of calcium, magnesium and potassium, when compared with the United Kingdom's Reference Nutrient Intake.

**Conclusion:**

This first large scale study of individual-based dietary intakes in the general population in Eastern Europe implies that intakes of saturated fat, sugar and complex carbohydrates are a cause for concern. The development of country-specific nutritional tools must be encouraged and nutritional campaigns must undergo continuing development.

## Background

After World War II, significant improvements in health developed across Europe. By the 1970s, however, while life-expectancy in the West had continued to rise, in Central and Eastern Europe (CEE) and in the Former Soviet Union (FSU), life expectancy began to plateau, or more worryingly, decrease. This gap in life expectancy, still evident today, is mainly due to mortality from circulatory diseases [1-3]. Available data suggest classical risk factors account for only a part of this difference between populations; it is therefore likely that other risk factors are involved [2,4].

A large number of dietary factors have been linked to cardiovascular disease (CVD) and other chronic diseases. For example, low consumption of fruit and vegetables and high intakes of saturated fat have been linked with increased CVD risk [5,6]. Both low intake of fruit and vegetables and high intake of saturated fat have been shown to be common in CEE/FSU [7-10]. Even though diet has been strongly implicated in the CVD epidemic in CEE/FSU, most of the available data are ecological; there are very few individual-based studies of nutrient intake in the region [11-14]. The lack of individual-level studies is exacerbated by the limited and often inconsistent information on the composition of foods (including traditional dishes), along with limited expertise in population-based assessment of diet.

The HAPIEE (Health, Alcohol and Psychosocial factors in Eastern Europe) study has been set up to investigate the effect of classical and non-conventional risk factors for vascular diseases in selected countries of CEE and FSU [15]. In this report, we present the dietary data assessed using a food frequency questionnaire (FFQ) in Russia, Poland, and the Czech Republic, and we compare the dietary intakes in these populations with those recommended by the World Health Organisation (WHO) and by the United Kingdom Department of Health.

## Methods

### Study populations and study subjects

The rationale and design of the HAPIEE study has been published previously [15]. In summary, the study was set up to investigate the role of alcohol, social and psychosocial factors and nutrition in CEE/FSU populations. The study consists of three urban cohorts in Novosibirsk (Russia), Krakow (Poland), and in six urban centres in the Czech Republic (Havířov/Karviná, Jihlava, Ústí nad Labem, Liberec, Hradec Králové, Kromeříz.). A fourth country, Lithuania was included subsequently, but FFQ data was not collected from this population. Random samples of men and women aged 45-69 years at baseline were selected from population registers in Poland and the Czech Republic, and from electoral lists in Russia. The study received ethical approval from the UCL/UCLH joint research ethics committee and from ethical committees in each participating country. All participants gave written informed consent. A total of 28,947 subjects (overall response rate 59%) were recruited. The baseline survey was conducted in 2002-2005 and involved completion of structured questionnaires and an examination in clinic. The questionnaires covered health, medical history, health behaviour, socioeconomic circumstances, psychosocial factors and diet. All questions were translated from English into each language and back translated into English to check for accuracy. In Russia and Poland, questionnaires were completed in a clinic, whereas in the Czech Republic, the subjects self-completed the questionnaires at home. This difference is reflected by a higher percentage of Russians and Poles in our sample with complete data (99.9% and 87%, respectively) compared with Czechs (82%).

### Dietary assessment

Diet during the preceding three months was assessed using a FFQ. The FFQ was based on the version used in the Whitehall II Study [16] which in turn was adapted from the original instrument developed by Willett [17]. The Czech, Russian, and Polish FFQs consisted of 136, 147,148 food and drink items, respectively; the different numbers of questions are due to country-specific dishes. A country-specific portion size for each food was specified, and participants were asked how often, on average, they had consumed that amount of the item during the last three months, with nine responses ranging from "never or less than once per month" to "six or more times per day". Frequency of food consumption was converted into daily food consumption, and daily intakes of 42 nutrients were then calculated by multiplying the frequency of food consumed per day by the nutrient content of the specified standard portion. This process included the following steps:

#### 1. Food matching

The collaborators in each country were asked to confirm the food names and portion sizes for foods listed in their FFQ.

#### 2. Collection of country-specific information

The collaborators provided the source of food composition data (FCD) available in their country along with an English translation of each country's nutrient definitions. Clarification was also sought on whether or not FCD represented raw or cooked foods and had added salt or fat. Due to time constraints, FCD was only investigated for foods most commonly consumed (on average ≥2-4 times/week, e.g. borsch), and composite food items (e.g. beef - roast, steak, stew). For composite food items, the local nutritionists estimated food composition based on weighted nutrient values according to frequency of consumption (i.e. the most commonly consumed beef dish eaten in a country would contribute more weight to the overall food composition values for beef).

#### 3. Compilation of data

As country-specific food composition tables were not entirely comparable (e.g. due to different analytical techniques), the McCance & Widdowson tables, which are the most comprehensive FCD available to date, were used as the main source of FCD [18]. The McCance & Widdowson tables contain FCD for at least 92% of the food items assessed in this study and were also used in the calculation of FCD from recipes (two items). For the additional, mainly country-specific foods, we used the local food composition tables (eight items in Poland and two items in Russia) [19,20]. In addition, we also used the United States Department of Agriculture (USDA) Nutrient Database (one item) [21]; the International table of glycemic index and glycemic load values (maximum 76% of items) [22]; and manufacturer data (one item). Data related to the additional questions at the end of the FFQ i.e. milk type, consumption of visible fat, and foods eaten repeatedly more than once per week, which were not listed in the FFQs, were also used in the compilation of the FCD.

#### 4. Reconciling discrepancies

Any discrepancies in the definition of nutrients between countries were dealt with accordingly either by estimating values based on a similar food in the McCance & Widdowson tables (e.g. values for fatty acids for traditional Russian foods, as these values were not available in the Russian tables) or by recalculation (e.g. the folate values in the USDA database includes folic acid but, as folic acid fortification has not been introduced in the countries of interest, the folic acid value was subtracted from total folate).

5. Finally, a *visual cross-check *was completed after food composition data was entered for each food to search for data editing errors. FCD for items present in two or more of the databases were once again cross-checked for data editing errors.

### Exclusions of subjects

A number of subjects had to be excluded from the final analysis. First, we excluded 512 subjects with missing data on basic socio-demographic data and anthropometry. Second, we excluded 676 subjects with missing values for more than 15 FFQ questions. Third, we excluded 610 subjects who answered 'No' to the question "Are the foods and drinks listed in the previous table representative of the foods and drinks that you consumed in the last 3 months?" but did not give details of any other foods that are eaten more than once a week (energy intakes among these subjects were significantly lower than in subjects included in the final analysis).

Fourth, we excluded 264 subjects with extreme nutrient values, identified on the basis of predicted energy expenditure [23]. To do so, we estimated the ratio of energy intake (EI) to basal metabolic rate (BMR) [24], subjects above and below 0.5% of EI/BMR were considered as reporting extreme values; this reduced the amount of extreme energy values without losing too much data.

Finally, after excluding the subjects with extreme energy intakes, the frequencies reported by the 50 subjects with the 10 highest values for either energy or carbohydrate or protein or fat or alcohol were examined. As a result, a further 15 subjects were excluded from the analyses as they had between one and four FFQ items which were implausibly high and the remaining FFQ items were also not in line with expectations. To ascertain whether this approach of identifying outliers was too conservative, we examined the number of subjects with energy intakes <1000 kcal/day or >5000 kcal day. The majority of subjects with energy intakes <1000 kcal day (n = 344), had sedentary occupations while the majority of those with energy intakes >5000 kcal day (n = 127) were involved in physical labour; the intakes were therefore considered plausible.

### Statistical analysis

After exclusions, a total of 26,870 subjects were available for the final analyses (7,913 Czechs, 9,098 Russians, and 9,859 Poles). First, we estimated crude and age-adjusted means of nutrient intakes by sex and country. Second, energy-adjusted nutrient intake was calculated using a regression model with absolute nutrient intake as the dependent variable and total energy intake as the independent variable [25]. The energy-adjusted intake is the sum of the residual and the expected intake of a given nutrient at the mean energy intake of the study sample. Finally, we estimated the proportion of subjects who did and did not comply with healthy diet recommendations (see below). Stata version 8.0 was used for all analyses [26].

### Diet quality

Diet quality was assessed by comparing selected food groups and nutrient intakes to two external dietary recommendations, one based on foods and macro-nutrients and one based on micro-nutrient intake.

First, the Healthy Diet Indicator (HDI) was based on the WHO recommendations for the prevention of chronic disease [27]. A dichotomous variable was generated for each food group or nutrient intake included in the recommendations, similar to those presented previously - percentage contribution to energy from saturated fats, polyunsaturated fats, protein, complex carbohydrates, and free sugars, along with consumption of fruit and vegetables (g/day) and pulses and nuts (g/day) [28,29]. In the current study, the HDI is the sum of seven dichotomous variables, each coded as 0/1, so that each subject will have a score value ranging from 0 to 7. From the full HDI, we omitted total fat and carbohydrate (to avoid overlap between categories) and salt (it was not known how much salt was added during preparation of meals or at the table). Intakes of macronutrients are presented as a percentage of energy intake without energy provided by alcohol (because alcohol consumption differed considerably between countries).

Second, the micro-nutrient intake in the cohorts was compared with the United Kingdom's Recommended Nutrient Intake (RNI). The RNI is defined as "the amount sufficient or more than sufficient to meet the nutritional needs of practically all healthy persons in a population" (Committee on Medical Aspects of Food Policy [30].

## Results

Characteristics of the subjects are presented in Table 1. Age, body mass index (BMI), and employment status were similar across the populations studied, except for the relatively lower BMI among Russian men, and higher BMI among Russian women, which is consistent with previous studies [31]. The ratio of energy intake to BMR indicates potential under-reporting, particularly among the Czech and Polish males, and less so among the Russian subjects. Similarly to earlier data, smoking prevalence was highest among Russian men and lowest in Russian women [32]. Approximately one-third of Russians and Poles in the sample hold a University qualification in this study; the proportion was lower among the Czech sample.

**Table 1:** Characteristics of subjects by country and sex; mean and standard deviations (SD) or percentages

	Czech		Russia		Poland	
	**male**	**female**	**male**	**female**	**male**	**female**

n-size	3690	4223	4135	4963	4815	5044

						

Age years	58 (7)	58 (7)	58 (7)	58 (7)	58 (7)	57 (7)

**Anthropometry**						

BMI (kg m^2^)	27.8 (3.7)	27.3 (5.0)	26.5 (4.3)	29.8 (5.6)	27.1 (3.8)	26.9 (4.7)

Ratio of total energy intake: BMR	1.1 (0.4)	1.4 (0.5)	1.6 (0.5)	1.6 (0.5)	1.3 (0.4)	1.5 (0.5)

**Smoking (%)**						

Smokers	30	24	50	10	36	29

Ex-smoker	38	22	24	4	36	20

Never	31	54	26	85	28	51

**Employment status (%)**						

Supervisor/manager	26	16	26	19	28	19

Employee, without inferiors	54	73	69	77	51	68

Self-employed	20	10	4	2	21	13

**Education (%)**						

None or primary	6	17	11	9	9	14

Vocational	44	30	22	30	28	15

Secondary	32	42	35	33	33	44

University	18	10	32	26	30	27

Absolute energy intakes and energy-adjusted nutrient intakes are presented in Table 2. Russian subjects had the highest mean energy intake (males 11.7 MJ/day, standard deviation (SD) (3.5); females 9.9 MJ/day, SD (2.9)), while the Czech sample had the lowest (males 8.7 MJ/day, SD (3); females 8.2 dMJ/day, SD (3)). In fact, the Russian sample had the highest intakes of most nutrients, apart from vitamin C and alcohol of which Czech men had the highest intakes, and NSP, folate, calcium, magnesium and potassium of which Polish subjects had the highest intakes. Table 3 shows that the energy contribution from protein was similar among all three countries. Whereas Czech and Polish subjects obtained most of their energy from carbohydrates, it is evident that total fat was the main source of energy among Russian subjects, with most of their energy being provided by monounsaturated fat.

**Table 2:** Absolute energy intakes and energy-adjusted nutrient intakes among males and females - means and standard deviations (SD)

	Absolute intakes						Energy-adjusted intakes^±^					
	**Czech**		**Russian**		**Polish**		**Czech**		**Russian**		**Polish**	

***Males***												

n-size	3690		4135		4815		3690		4135		4815	

	mean	s.d	mean	s.d	mean	s.d	mean	s.d	mean	s.d	mean	s.d

Energy (MJ)	8.7	3.0	11.7	3.5	9.5	2.9	-	-	-	-	-	-

Total fat (g)	85	35	133	49	94	36	85	13	132	17	94	15

Saturated fat (g)	31	13	46	20	38	15	31	6	45	8	38	7

Monounsaturated fat (g)	30	13	48	20	32	13	29	6	48	7	32	7

Polyunsaturated fat (g)	15	7	25	10	13	5	15	3	25	7	13	3

Protein (g)	92	34	120	38	103	31	92	15	121	19	104	14

Total carbohydrate (g)	230	89	278	83	264	86	230	40	280	43	264	39

Sugars (g)	107	54	121	48	121	56	106	35	121	32	120	34

Starch (g)	122	52	153	48	140	46	122	32	154	35	141	32

Non-starch polysaccharide (g)	17	8	18	6	19	7	17	6	18	5	19	5

Vitamin C (mg)	160	135	111	97	147	94	160	107	110	86	148	78

Vitamin E (mg)	8	4	13	5	8	3	8	2	13	3	8	2

Folate (μg)	294	115	300	101	322	118	295	68	301	61	323	79

Carotenes (μg)	5310	4026	10833	5082	7779	4544	5331	3522	10999	5113	7867	4182

Iron (mg)	11	4	15	4	14	4	11	2	15	2	14	2

Calcium (mg)	721	362	811	411	872	425	721	250	806	289	867	282

Magnesium (mg)	278	97	286	91	294	94	280	43	287	44	295	45

Potassium (mg)	3280	1259	3623	1160	3774	1360	3288	613	3640	628	3783	755

Alcohol (g)^±±^	13	17	10	17	4	9	15	17	13	17	7	10

												

***Females***												

n-size	4223	4963	5044	4223	4963	5044	4223	4963	5044	4223	4963	5044

Energy (MJ)	8.2	3.0	9.9	2.9	8.7	2.6	-	-	-	-	-	-

Total fat (g)	79	33	114	40	81	31	79	13	113	15	80	13

Saturated fat (g)	29	13	38	16	33	14	29	5	37	6	33	7

Monounsaturated fat (g)	27	12	41	15	27	11	27	6	41	7	26	6

Polyunsaturated fat (g)	15	6	24	10	11	5	15	4	24	7	11	3

Protein (g)	85	32	102	33	93	28	86	14	102	17	93	13

Total carbohydrate (g)	239	99	245	80	258	84	238	37	246	39	258	35

Sugars (g)	126	69	119	48	128	59	125	39	119	31	127	35

Starch (g)	110	51	122	46	127	43	110	30	122	35	128	31

Non-starch polysaccharide (g)	20	12	18	7	19	8	20	7	18	5	19	5

Vitamin C (mg)	229	242	128	119	163	109	224	165	128	107	163	91

Vitamin E (mg)	9	4	13	5	8	3	9	3	13	3	8	2

Folate (μg)	303	135	274	100	321	124	304	77	276	70	322	85

Carotenes (μg)	6664	5161	11533	5620	8398	5020	6672	4427	11711	5637	8490	4610

Iron (mg)	11	5	13	4	13	4	11	2	13	2	13	2

Calcium (mg)	835	411	769	363	899	438	835	264	769	277	895	287

Magnesium (mg)	283	112	256	84	284	92	284	48	257	45	285	43

Potassium (mg)	3589	1621	3359	1128	3729	1355	3585	788	3377	678	3736	760

Alcohol (g)^±±^	3	6	1	3	1	4	5	7	2	4	3	6

^± ^Nutrients were log and then anti-log transformed for energy adjustment using the residual method.

^±± ^Sample sizes were as follows: Czech male (n = 3267); Czech female (n = 2691), Russian male (n = 3322); Russian female (n = 3152); Polish male (n = 3179); Polish female (n = 1737).

**Table 3:** Percentage contribution of macronutrients and alcohol to energy intake by country and sex

Macronutrient	Czech			Russia			Poland		
	**Total**	**Males**	**Females**	**Total**	**Males**	**Females**	**Total**	**Males**	**Females**

	**7913**	**3690**	**4223**	**9098**	**4135**	**4963**	**9859**	**4815**	**5044**

Protein %	18	18	17	17	17	17	18	18	18

Carbohydrate %	43	41	45	39	38	39	45	44	47

Total fat %	36	36	36	43	42	43	36	37	35

Saturated fat %	13	13	13	14	14	14	15	15	14

Monounsaturated fat %	13	13	12	16	16	16	12	13	11

Polyunsaturated fat %	7	6	7	9	8	9	5	5	5

Alcohol %	3	5	1	1	3	0.4	0.9	1	0.4

**Figure 1:**
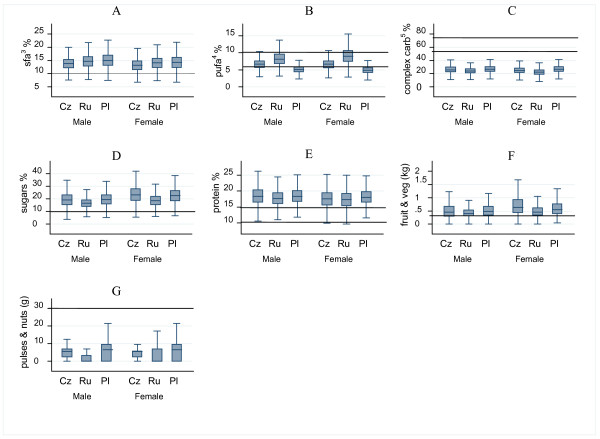
**Median intakes and inter-quartile ranges^1 ^of the Healthy Diet Indicator (HDI) components among subjects^2^**. ^1^Percentages denote contribution to non-alcohol calorie intake; line across graph denotes WHO recommendation. ^2 ^Czech Republic (CZ); Russia (RU); Poland (PL). ^3^saturated fatty acids; ^4^polyunsaturated fatty acids; ^5^complex carbohydrates.

The mean HDI scores were lower among the Polish males (1.0, SD = 0.7) and females (1.0, SD = 0.7), compared to Czech males: 1.4, SD = 0.8; Czech females: 1.7, SD = 0.8; Russian males: 1.4, SD = 0.8; and Russian females: 1.5, SD = 0.8. Table 4 and Figure 1 show the percentage of subjects meeting current dietary recommendations, and the median intakes of each component, respectively. Comparison of dietary intakes with the Healthy Diet Indicator (HDI) shows that very few subjects consume the recommended levels of SFA, complex carbohydrates, sugars, and pulses or nuts (Table 4). Approximately two-thirds of Czech (63%) and Russian subjects (64%) reported consuming the recommended levels of polyunsaturated fatty acids, while only less than one-fifth (17%) of the Polish sample met this recommendation. Few subjects from the Czech Republic (14%), Russia (17%), and Poland (8%) reported consuming the recommended protein intakes; most of them consumed too much (Figure 1).

**Table 4:** Percentage of HAPIEE subjects (aged 50+) meeting the WHO recommendations and the RNI^↕ ^for micronutrients

Component	Recommended intake	Percentage meeting recommendation					
		**Czech**		**Russia**		**Poland**	

		**male**	**female**	**male**	**female**	**male**	**female**

***WHO recommendation***							

*n-size*		**3690**	**4223**	**4135**	**4963**	**4815**	**5044**

		**%**	**%**	**%**	**%**	**%**	**%**

Saturated fat %^↕↕^	0-10	5	10	4	5	3	6

Polyunsaturated fat %^↕↕^	6-10	58	62	69	61	17	15

Protein %^↕↕^	10-15	11	17	13	20	7	9

Complex carbohydrates %^↕↕^	55-75	0.1	0.02	0.02	0	0.04	0.05

Free sugars %^↕↕^	0-10	4	1	6	2	3	1

Fruit and vegetables (g)	≥400	58	77	49	60	63	73

Pulses & nuts (g)	>30	2	2	2	3	3	2

							

***RNI***							

*n-size*		**3106**	**3463**	**3477**	**4074**	**3970**	**4051**

		**%**	**%**	**%**	**%**	**%**	**%**

Vitamin C (mg)	≥40	97	97	92	92	98	98

Folate (μg)	≥200	80	81	86	78	89	86

Calcium (mg)	≥700	43	57	51	48	59	64

Iron (mg)	≥8.7	71	66	95	48	91	84

Magnesium (mg)	≥300 (men), ≥270 (women)	33	45	35	36	39	48

Potassium (g)	≥3.5	33	42	46	37	50	48

^↕ ^The Reference Nutrient Intake (RNI) for 50+ year olds.

^↕↕ ^Percentages denote contribution to non-alcohol calorie intake.

One food group, of which a reasonable percentage of the cohort (64%) appeared to have consumed the recommended levels, was fruit and vegetables (Table 4). A higher percentage of females (70%) than males (57%) met this recommendation. As seasonality, in particular, may influence fruit and vegetable consumption, the season in which the FFQ was completed was considered (not shown in table). The highest percentage of subjects who met the recommendation were those who completed the FFQ in the Autumn - Czech males and females (60% and 81%, respectively), Russian males and females (75% and 81%, respectively), and Polish males and females (70% and 78%, respectively). On the other hand, few Russian males who completed the FFQ in Winter, Spring, and Summer met the recommendations (48%, 40%, and 38%, respectively), and only 38% of Russian females who completed the FFQ in Summer reported a daily consumption of more than 400 g of fruit and vegetables.

As the majority of the subjects are aged 50 years and older (82%), Table 4 presents the percentage of those aged 50 years and older meeting RNIs for this age category. The majority of subjects consumed the required amounts of vitamin C and folate, but fewer subjects consumed adequate amounts of calcium, magnesium and potassium, according to the RNI guidelines.

## Discussion

This study is one of the first individual-level dietary studies in Eastern Europe, and the largest one which examined general population samples. We found plausible levels of nutrient intakes and in line with expectations, a large proportion of subjects did not meet healthy diet recommendations. The percentage contribution towards energy from saturated fats and sugars are worryingly high, whereas the percentage contribution towards energy from complex carbohydrates and intakes of calcium, magnesium and potassium, pulses and nuts were lower than that recommended. This study demonstrates the feasibility of large scale nutritional studies in Eastern Europe, but it also highlights the practical difficulties, including the lack of reliable and comparable local food composition data.

### Limitations of the study

One potential limitation of this study is the chosen method of dietary assessment. The FFQ method has been criticised [33], mainly because it does not cover all range of foods and because it misclassifies real portion sizes and thus either underestimates or overestimates energy and nutrient intakes [16,34]. However, FFQs are designed to establish habitual intake and it was not feasible in our study to use a dietary diary in such large numbers. Diet in the preceding three months was assessed in this study therefore it may not be wholly representative of usual intake.

The second issue, related to dietary methods in general, is the potential under-reporting of intakes [35]. Our data on energy intake and BMR indicate under-estimation of intakes, and this might be due to the method of FFQ administration. In general, intakes were higher among the Russian and Polish samples, where FFQs were completed under supervision; in contrast, the intakes among Czech subjects were generally lower, which may reflect the fact that the FFQ was completed unsupervised at home. However, while this would make absolute intakes not directly comparable across populations, the data should allow comparison of energy adjusted intakes; within-country analyses should also be unbiased.

Third, local but internationally comparable food composition tables do not exist for these countries. There are efforts to harmonise European food composition data and to develop food composition databases for Europe, including Poland, but such a databank is currently unavailable [36]. To overcome the lack of local composition tables, we have, in essence, used the McCance & Widdowson tables as the 'central' food tables, with inclusion of information about country-specific foods. While this approach may have introduced some misclassification, we believe that it is far preferable to using existing local food tables which have often questionable origin and which often differ considerably in nutrient composition of identical foods due to differences in composition, analytical methods and modes of expression.

Fourth, the response rates were 55% in the Czech Republic to 61% in Poland and Russia; since non-respondence is often associated with health status and health behaviours, it is possible that our results show a more favourable picture than if truly representative samples were examined. Similarly, as the centres involved in this study were located in urban areas only, we were not able to examine nationally representative samples. Although the levels of, and trends in mortality and health behaviours in our study centres are similar to national figures, and differences in health and behaviours between countries in our study reflect differences observed at the national levels, it would be imprudent to claim that our results apply to the whole population. Also, although the Czech cities involved in our study do not differ dramatically to Prague, they are smaller than Krakow and Novosobirsk and it could be argued that city size may influence diet, thus introducing a potential bias to the study.

### Consistency with earlier studies

As this is the first large-scale analysis of nutrient intakes in these countries, there are few published studies to which the results of this current study can be compared [11-14]. Nevertheless, it is useful to compare our results with other available data.

The lowest mean energy intakes among the countries studied were found in the Czech sample. Our estimates are also slightly lower than those produced by the agricultural ministry in the Czech Republic (12 MJ/day) [13]. Though the Czechs in this study gained most of their energy from carbohydrates (43%), this contribution was lower (62%) and the contribution from protein (18%) and total fat (36%) was higher than reported previously (12% and 26%, respectively) [13].

In the Russian sample, the median energy intake observed amongst males in this study was similar to that found from the 24-hour recall in the Russian Longitudinal Monitoring Survey (RMLS) [11]. By contrast, women in our study had a higher energy intake (9.8 MJ/day) than in the RLMS (7.1 MJ/day); however, the proportion of obese women in our study (45%) was also higher than in the RMLS (20%) [11]. Although Russian men had the highest energy intakes in this present study, they had the lowest BMI; this is similar to a previous study which found that though BMI was significantly lower among Russian men compared to men from the United States, Russians had a significantly higher energy intake (kcal/kg body weight) [37]. As half of Russian men smoke, and as nicotine has been shown to increase energy expenditure and fat oxidation, smoking may partly explain why Russian men have the highest energy intakes, yet the smallest BMI compared to Czech and Polish men [38,39]. Occupation may also have an influence on energy intake - of those who provided details on employment (54%), Russian men were mostly involved in physical occupations (43%), whereas the majority of Czech and Polish men were involved in sedentary occupations (45% and 50%, respectively).

Using a FFQ, Zaridze and colleagues assessed the diets of 217 cases with colorectal cancer and 217 controls from Moscow and Khabarovsk [14]. Daily median intakes of protein, polyunsaturated fatty acids and starch among control subjects were similar to our study. However, the intakes of total fat, saturated fat, monounsaturated fat and some micro-nutrients, including vitamin C, were higher among controls compared to our study. It was the intake of beta-carotene, however, which differed most between the two studies, with the median daily intakes in this present study (11.8 μg for males, 11.9 μg for females) being more than five times higher than those found in the Zaridze study (2 μg for males, 2.3 μg for females).

In Poland, a study of 4,440 men and women who completed 24-hour recalls [12] reported similar total energy intakes to those found in our study; on the other hand, estimates by the Polish agricultural ministry (14 MJ/day) [13] are higher than in our study. The contribution to energy from total fat, and mean intakes of carbohydrates and saturated fats among our Polish subjects are similar to earlier reports (35%, 265 g/day and 39.5 g/day, respectively) [12]. The most considerable difference in intakes between our Polish cohort and other reports are the protein intakes. Both protein intakes and it's contribution to energy was much higher among our Polish cohort (mean 98 g/day and 18%, respectively) compared to other reports (mean 68 g/day and 10%, respectively) [12,13].

The above comparisons with previous studies must be treated with caution. First, the designs of the studies differ, with intakes generally based on whole samples rather than distinguishing between intakes of males and females. Research has shown that there are gender differences in nutrient intake and dietary patterns, suggesting that male and female intakes should be studied separately [40,41]. Secondly, different tools were used to measure dietary intake e.g. Serra-Majem and colleagues report Polish intakes derived from 24-hour diet recalls but this method provides only a snap-shot of a person's diet and may not be representative of habitual diet [12]. The data produced by the Polish and Czech agricultural ministries were based purely on estimations, and thus must also be treated with caution [13].

Third, different food composition tables can differ dramatically [18,19]. In a pilot study, we have previously found huge (more than 10-fold) discrepancies in compositions of identical foods, particularly in relation to micro-nutrients (unpublished). As regards the intakes in the Russian case-control study [14], the FFQ development and the reliability of the Russian FCD published in 1987 is unclear [14,42]. Finally, the above studies report intakes which were assessed a decade or more ago (e.g. the study by Zaridze *et al *collected data not long after the social crisis which occurred following the 1991 dissolution of the FSU [14]). Twenty years later, diets may well have changed. For instance, following price liberalisation, prices of animal products rose dramatically while fruit and vegetables became widely available and cheap [43]. Similarly, the relatively high intake of protein among our Polish subjects may partly reflect improved affordability of meat over the last decade.

### Quality of diet

The HDI has recently been described as "a very strong good tool for assessing the quality of diet" [44]. Our results indicate that diet in this region is still a cause for concern. The mean HDI score in the study was lower than that previously reported in Europe (mean HDI scores = 2.5-3.4) and the United States (mean HDI score = 3.4) [45]. The energy contribution of saturated fat was substantially higher than recommended; only a meagre 4% of men and 7% of women in the cohort obtained 10% or less of their energy from saturated fat. The high intakes of saturated fat could be inherited from the original guidelines in the FSU, which recommended high intakes of protein in order to maintain good health. In doing so however, intakes of saturated fat are liable to increase as high animal protein diets are also likely to be high in saturated fat. This could also be responsible for the large contribution to energy from protein in the other two countries.

One of the most startling differences in intake between countries is the significantly lower proportion of Polish subjects (16%) meeting the recommended intakes for polyunsaturated fats compared to the Czech (60%) and Russian subjects (65%). This is surprising, given the decrease in saturated fat intakes and increase in polyunsaturated fats (by a substantial 57%) in Poland between 1990 and 1999 [46]. These changes were a result of the lower subsidies for animal fat products and the increased demand for vegetable fat, in particular for rapeseed and soya oil. Despite this increased demand, the median daily consumption of vegetable oil is much lower among our Polish cohort (1.2 ml/day) compared to our Czech (4.7 ml/day) and Russian cohort (11 ml/day).

The proportion of energy provided by sugar in this cohort is also worrying. A diet high in sugar has been recently shown to increase CHD risk [47], and high glycaemic load seems to increase C-reactive protein (CRP) concentrations which, in turn, may lead to increased CVD risk [48,49]. On the other hand, the contribution to energy from complex carbohydrates was substantially lower in this study compared to the WHO recommendations of 55-75% [27]. This is of added concern as complex carbohydrates have been implemented in the prevention of chronic diseases including CVD [50].

Approximately two-thirds of the cohort reported consuming the WHO recommended intakes for fruit and vegetables (≥400 g), except for Russian men, of whom less than half were consuming more than 400 g of fruit and vegetables. The Russian diet has long been characterised by low fruit and vegetable consumption mainly due to the short growing season and the priority placed on increasing consumption of animal protein [43]. In fact, it has been suggested that the decline in fruit and vegetable consumption could explain 28% of the increase in CVD mortality in Russia between 1992 and 1994 [43]; although some argue that changes in CEE mortality rates are not so attributable to diet [51]. The mean consumption of pulses and nuts in our cohort was one-fifth of the recommended 30 g/day.

The majority of subjects in our study appear to be consuming sufficient quantities of vitamin C, folate and iron. On the other hand, few subjects reported consuming sufficient quantities of calcium, magnesium, and potassium. While intakes of calcium are not as strongly associated with CVD risk, calcium has been advocated in lowering the risk of developing other diseases, such as osteoporosis and cancer [52,53]. On the other hand, both dietary potassium and magnesium have been associated with heart disease. High intakes of potassium have been associated with lower blood pressure and lower rates of stroke, yet the effect on CVD mortality in Western societies remains unclear [54-56]. There does, however, appear to be clearer evidence suggesting a protective effect of dietary magnesium on CVD risk [57,58]. The results from this current study therefore suggest that the consumption of foods such as fruit, vegetables, pulses and nuts should continue to be encouraged among these populations.

Indeed, since the dissolution of the FSU, improvements in diet have been promoted and positive changes are evident in the countries studied. In Poland, between 1989 and 2000, there was a significant decrease in consumption of animal fats and milk, and an increased consumption of vegetable fats and fruit [59]. It has been suggested that such changes were responsible for the decrease in CVD mortality in Poland after 1991 [59]. Encouraging trends are also evident in the Czech Republic, where following health promotion activities and the abolition of subsidies for certain products, consumption of beef, pork and butter fell, and consumption of chicken, fish and vegetable oils increased between 1989 and 1997 [60]. During the same time period, total serum-cholesterol concentration decreased substantially after 1988 and it has been suggested that these changes played a role in decreasing mortality rates in the country during the 1990s [60]. In Russia, the push towards consumption of livestock products has been largely discontinued and consumption of high fat livestock products and sugar has been reported to have fallen [61]. Though as the same report stipulates, and as the results of the current study show, Russians appear to maintain traditional preferences for livestock products, in particular meat, poultry and dairy products.

It can be said that the above trends are favourable, particularly in the Czech Republic and Poland, and while it cannot be said that diet is wholly responsible for changes in health outcomes, it may play an important role in narrowing the gap in mortality between these countries and those in the West. The high-fat, high-sugar diets which exist among our cohort are not so favourable, and may contribute to the high prevalence of obesity and circulatory diseases reported in these countries [62-64]. Therefore, the results from our study indicate that food and nutrition policies must be supported and that replacing fats as the major source of energy intake with complex carbohydrates, such as fruits and vegetables, should be encouraged [65].

## Conclusion

This large-scale assessment of nutrient intakes in CEE/FSU is the first to show that such studies are feasible. We found that energy derived from saturated fat and sugar were well above recommendations and energy from complex carbohydrates, intakes of calcium, magnesium, potassium, pulses and nuts fell well below recommendations. Despite reported improvements in diet that have occurred in the region since the fall of communism around 1990, our results imply that existing health promotion campaigns and nutrition policies need to undergo further development.

## Abbreviations

(HDI): Healthy Diet Indicator; (WHO): World Health Organisation; (CEE): Central and Eastern Europe; (FSU): Former Soviet Union; (CVD): Cardiovascular Disease; HAPIEE: (Health, Alcohol and Psychosocial factors In Eastern Europe); (FFQ): Food Frequency Questionnaire; (FCD): Food Composition Data; (USDA): United States Department of Agriculture; (BMR): Basal Metabolic Ratio; (EI): Energy intake; (RNI): Reference Nutrient Intake; (BMI): Body Mass Index; (SFA): Saturated Fatty Acids; (PUFA): Polyunsaturated Fatty Acids; (RMLS): Russia Longitudinal Monitoring Survey; (Coronary Heart Disease): CHD; (CRP): C-Reactive Protein.

## Competing interests

The authors declare that they have no competing interests.

## Authors' contributions

SB was involved in the data analysis and writing of the manuscript. MB and HP offered advice on the statistical analyses and AW provided advice on the methods. OB, US, AJ and LM provided information on traditional food composition. All authors commented on drafts and approved the final text.

## Pre-publication history

The pre-publication history for this paper can be accessed here:

http://www.biomedcentral.com/1471-2458/9/439/prepub
